# Evaluation of Deodorizing Effects of *Saccharina japonica* in 10-Month-Old ICR Mice Using a Novel Odor Marker Associated with Aging

**DOI:** 10.1155/2022/1410144

**Published:** 2022-02-09

**Authors:** Ji Eun Kim, Yun Ju Choi, Su Jin Lee, Jeong Eun Gong, Ji Eun Seong, So Hae Park, Dae Youn Hwang

**Affiliations:** ^1^Department of Biomaterials Science (BK21 FOUR Program), College of Natural Resources & Life Science/Life and Industry Convergence Research Institute, Pusan National University, Miryang 50463, Republic of Korea; ^2^BK21 FOUR Program Team, Department of Biomaterials Science, College of Natural Resources and Life Science, Pusan National University, Miryang 50463, Republic of Korea

## Abstract

The potential deodorizing effects of *Saccharina japonica* have been evaluated by determining their deodorizing performance, but they are yet to be validated in experimental animals. The deodorizing effects of *S. japonica* were examined in an animal model using a novel odor marker associated with aging by comparing the concentration of odor component in urine obtained from two- and 10-month-old ICR mice using gas chromatography-mass spectrometry (GC-MS), and the changes in the trimethylamine (TMA) concentration, ammonia level, and structure of sweat gland were determined after exposing 10-month-old ICR mice to 70% ethanol extract of *S. japonica* (EESJ) for four weeks. *In vitro* analysis was performed to confirm the composition of EESJ with respect to the total flavonoid contents (TFC, 28.6 ± 2.5 mg/g), total polyphenol contents (TPC, 107.3 ± 8.9 mg/g), and total condensed tannin contents (TTC, 65.7 ± 5.2 mg/g) contents, as well as to the deodorizing performance to ammonia and acetic acid (91.2 ± 7.8% and 54.8 ± 6.3%, respectively). *In vivo* analysis revealed TMA to be the novel odor marker associated with aging among the 19 odor components evaluated, considering the higher concentration in the urine of 10-month-old ICR mice. The peak area of TMA on the gas chromatogram was significantly lower in the 10-month-old ICR mice treated with EESJ than in the two-month-old mice. A similar decrease was observed in the level of ammonia obtained from the dirty bedding of the EESJ-treated group. Moreover, tissues obtained from the mouse foot of the group exposed to EESJ showed a dose-dependent decrease in the gland tube number of sweat glands and the TMA dehydrogenase transcription level. Overall, these results provide novel evidence that the administration of EESJ helps reduce the body TMA and ammonia concentrations, resulting in reduced odor and a decrease in the number of sweat glands and the expression of TMA dehydrogenase in the ICR mouse feet.

## 1. Introduction

Body odor in humans is determined by various factors, including the genetic background, physiological conditions, behavioral patterns, food ingestion, and disease types [1]. Despite the diversity of influencing factors, the major contributor is the bacterial activity in skin gland secretions [2]. Numerous skin bacteria, including *Corynebacterium jeikeium*, *Staphylococcus haemolyticus*, *Staphylococcus hominis*, and *Staphylococcus epidermidis*, help produce odorant substances [3, 4]. Most of the chemical compounds required by the skin bacterial flora are secreted from the apocrine sweat glands on the skin, which are then metabolized into odorant substances [2]. To date, three types of odorant substances have been identified as the main compounds in the human axilla. The first two key odoriferous steroids identified were androstenone (5*α*-androst-16-en-3-one) and androstenol (5*α*-androst-16-en-3*α*-ol), even though their detectable levels in human sweat are extremely low [5]. E-3M2H (E-3-methylhex-2-enoic acid) and HMHA (3-hydroxy-3-methylhexanoic acid) were the second principles identified. HMHA is more abundant than E-3M2H and is quantitatively the most dominant human odorant [6, 7]. The diverse class of E-3M2H and HMHA in sweat is represented as branched or unbranched, saturated, unsaturated, or hydroxylated acids [7, 8]. Present in minute quantities, a different class with carbon skeletons (sulfanylalkanols) have been detected as the third class of odorants in axillary secretions [9]. Moreover, several amino acid conjugates, such as 3M2H-Gln, HMHA-Gln, Cys-Gly, and Cys, appear to be the key precursors for odorant acids. Three enzymes mediate the formation of these conjugates: zinc-dependent N*α*-acyl-Gln aminoacylase, zinc-dependent dipeptidase, and cystathionine *β*-lyase [9–12].

Among the skin odorants, several key markers showed a positive correlation between the amount of body odor and aging. The first identified human body odor component was 2-nonenal. The amount of 2-nonenal generated by the oxidative degradation of *ω*7 unsaturated fatty acid was significantly higher in the above 40 years of age group than in the below 40 years of age [13]. Other markers identified in nonaxillary skin odorants include dimethyl sulfone, benzothiazole, aldehyde, and nonanal, which were found to be more abundant in older subjects (41–79 years) than in younger subjects (19–40 years) [14]. Furthermore, the levels of pleasantness rating and intensity rating, which potentially detect body odors, were decreased or increased remarkably, respectively, in the old-aged group than in the middle-aged or young-aged groups [15]. Thus, identifying age-associated novel markers for body odor is essential for designing new deodorant ingredients and evaluating their effects. In particular, identifying an odorant marker in experimental animals is important because there are many limitations associated with time in a study involving aging humans.

Identifying deodorants and techniques to eliminate body odor has attracted considerable attention from scientists. Previous studies evaluated the deodorizing effects of various agents, such as deodorants, antiperspirants, disinfectants, underarm liners, triclosan, or special soaps with antiseptic plant extracts [16]. In particular, some natural products evaluated have provided excellent deodorizing effects. Champignon extract suppresses the generation of ammoniacal nitrogen in chicken liver homogenate and the blood concentration of indoleacetic acid and tryptamine in rabbits [17]. The quantity of methylthiol and allyl thiol decreased significantly after exposure to a mushroom (*Agaricus bisporus*) extract, while the mean malodor score was reduced after applying a supercritical hop extract in the clinical underarm odor reduction evaluation [18, 19]. Moreover, high deodorizing effects were detected using camelina (*Camelina sativa*) oil and immature pinecone extract [20, 21]. Furthermore, different fractions of the methanolic extract of *Salvia officinalis* (sage) reduced the axillary malodor level in healthy subjects compared with a control group [22]. The potential for the deodorizing effects of *S. japonica* was first provided with a previous study. The deodorizing performance was enhanced remarkably in the aqueous extract, acetone extracts, and ethanol extracts of *S. japonica* [23]. On the other hand, further animal studies are required to evaluate the deodorizing effects of *S. japonica*.

This study evaluated the deodorizing effects of ethanol extracts of *S. japonica* (EESJ) in old mice using a novel odor marker associated with aging to evaluate their potential as a new deodorant. These results provide important scientific evidence that TMA can be a vital odor marker associated with aging and that EESJ has potential deodorizing effects in 10-month-old ICR mice.

## 2. Materials and Methods

### 2.1. Preparation of *S. japonica* Samples and Extraction of EESJ

These methods were performed in accordance with a previously described study [24]. After purchasing *S. japonica* samples from Parajeju Com. (Jeju, Korea), they were deposited as voucher specimens (WPC-19-002) at the functional material bank of the Wellbeing RIS Center, Pusan National University. Dr. Jung Youn Park at the Biotechnology Research Division, National Fisheries Research and Development Institute, verified the *S. japonica* samples. First, the dried sample was ground to a powder using a pulverizer (MF-3100S; Hanil Electric Co., Seoul, Korea). The powder was mixed with 70% ethanol solvent at a fixed liquor ratio (1 : 10). An EESJ solution was extracted sequentially from the mixture at 25°C for 12 h using circulating extraction equipment (SHWB-30/45; Woori Science Instrument Co., Pocheon, South Korea). The total solution obtained through three extraction steps was passed through a 0.4 *µ*m filter and concentrated using a rotary evaporator (Eyela, Tokyo, Japan).

### 2.2. Quantification of Three Bioactive Compounds in EESJ

The concentration of three bioactive compounds in EESJ was quantified according to the basic principle described in a previous study [24]. First, the total phenolic contents (TPCs) were determined in EESJ using the Folin–Ciocalteu method [25]. After incubating a mixture of EESJ solution (1 mL) and Folin–Ciocalteu reagent (5 mL; Sigma-Aldrich Co., St. Louis, MO, USA) for 5 min, a 20% Na_2_CO_3_ solution (15 mL) was added to this mixture. The absorbance was measured repeatedly at 765 nm using a VersaMax plate reader (Molecular Devices, Pasadena, CA, USA). The final value of TPC in EESJ is presented as the gallic acid equivalent (mg) of the extract using a standard calibration curve of gallic acid (Sigma-Aldrich Co.).

The total flavonoid contents (TFCs) were measured in EESJ using the methods suggested in previous studies [26]. The EESJ sample (20 *µ*L) was mixed gently with 5% NaNO_2_ (60 *µ*L) and 10% AlCl_3_ (60 *µ*L) (Sigma-Aldrich Co.) and incubated at 25°C for 5 min. The absorbance of this mixture was determined using a VersaMax plate reader (Molecular Devices). The final concentration of TTC in EESJ was calculated from a calibration curve constructed using a purified (+)-catechin hydrate standard (Sigma-Aldrich Co.).

Furthermore, the total condensed tannin content (TTC) was determined in EESJ using the vanillin method [27]. After mixing the EESJ sample solution in 0.5 ml 80% m (Sigma-Aldrich) ethanol and the mixture solution (1% vanillin/MeOH and 8% HCL/MeOH, 1 : 1 ratio), the resulting mixture was incubated at 30°C for 20 min. The absorbance of this mixture was measured at 500 nm using a VersaMax plate reader (Molecular Devices). The final value of TTC in EESJ was determined using a standard calibration curve of a purified (+)-catechin hydrate standard Co.).

### 2.3. Measurement of Deodorizing Performance

The deodorizing performance of EESJ was measured using the methods described elsewhere [21]. This method suggested by the Korea Textile Inspection Testing Institute (KOTITI, Changwon, Korea) and the Japan Textile Evaluation Technology Council (JTETC) was performed in the gas detecting tube containing ammonia and acetic acid. In the tube (gas container (5 L) containing 3 L gas) with the sample fabrics (10 cm × 10 cm), the concentrations (*C*_*s*_, ppm) of ammonia and acetic acid were determined at 25°C for 2 h. The concentrations (*C*_*b*_, ppm) of ammonia and acetic acid were 100 and 50 ppm in the blank tube without the EESJ sample, respectively. The final value of the deodorizing performance was calculated as follows:

(1) $$\begin{array}{c} \text{Deodorization performance}\left(\%\right)=\left[\frac{\left(C_{b}-C_{s}\right)}{C_{b}}\right]\times 100. \end{array}$$

### 2.4. Animal Care and Experimental Design

The Pusan National University-Institutional Animal Care and Use Committee approved the animal experimental protocol for the deodorizing effect of EESJ (Approval No. PNU-2019–2297) based on ethical and scientific care guidelines. Male ICR mice at two and 10 months of age were obtained from the Samtako BioKorea Co. (Osan, Korea). All animals were provided with access to water and an irradiated standard chow diet *ad libitum* (Samtako BioKorea Co.). They were bred at 23 ± 2°C and 50 ± 10% relative humidity under a strict light cycle (lights on at 08 : 00 a.m. and off at 08 : 00 p.m.). The mice were housed in solid-bottom cages with wood shavings under specific pathogen-free (SPF) conditions. The Pusan National University-Laboratory Animal Resources Center is accredited by the Korea Ministry of Food and Drug Safety (MFDS; Accredited Unit 000231) and the Association for Assessment and Accreditation of Laboratory Animal Care (AAALAC) International (Accredited Unit 001525).

The novel marker associated with aging was identified from uncontaminated urine samples (Daejong Instrument Industry Co., Ltd, Seoul, Korea) from the two- and 10-month-old ICR mice (*n* = 5 each) bred individually for 24 h in metabolic cages.

**Figure 1:**
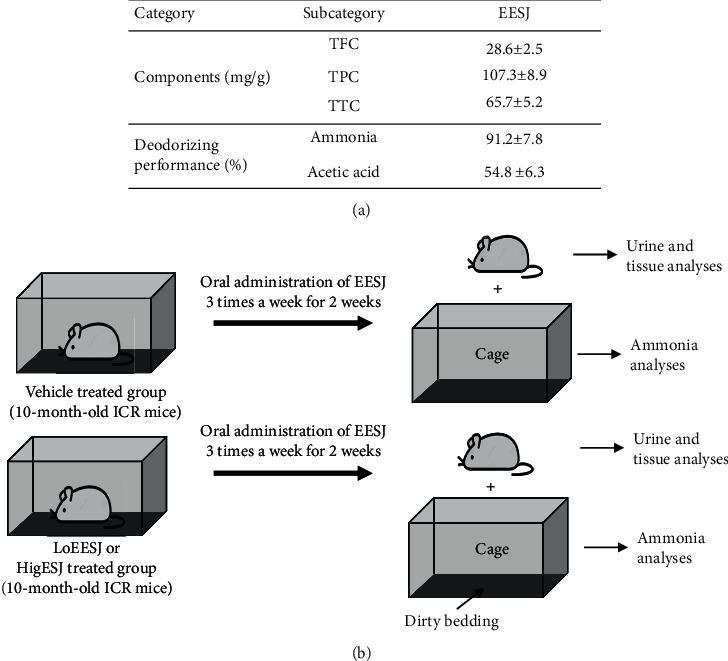
*In vitro* analysis and the experimental scheme for the animal study. (a) TFC, TPC, and TTC of EESJ were measured, as described in Materials and Methods. In deodorizing performance analysis, the changes in the concentration of ammonia and acetic acid gases were detected after 2 h in the bag 5 L containing the sample powders. The data are reported as the means ± SD. (b) 10-month-old ICR mice were administrated two doses of EESJ orally three times a week for two weeks. Subsequently, the changes in odor markers were analyzed in the urine, foot tissue, and dirty bedding. TFC, total flavonoid; TPC, total phenol; TTC, total condensed tannin.

The deodorizing effects of EESJ were evaluated by dividing the 10-month-old ICR mice (*n* = 21–24) randomly into one of the three groups (*n* = 7–8/group): (i) vehicle-treated group; (ii) low-dose EESJ treatment group (LoEESJ-treated group); and (iii) high-dose EESJ treatment group (HiEESJ-treated group). The LoEESJ-treated group was administered a single dose of 75 mg/kg body weight EESJ orally, whereas the HiEESJ-treated group received a single dose of 150 mg/kg body weight EESJ, every three days for two weeks. The vehicle-treated group was administered the same volume of 1× PBS under the same pattern. At 24 h after the final treatment, urine was collected from the metabolic cage of each mouse in all subset groups. The mice were then euthanized in a euthanasia chamber filled with CO_2_ gas, after which tissue samples were acquired and stored at −70°C in the Eppendorf tubes until assay. The dirty bedding was collected from the breeding cages on three, four, and five days before the final sacrifice, and the ammonia concentration was measured (Figure 1(b)).

### 2.5. Gas Chromatography-Mass Spectrometry (GC-MS) Analysis

GC-MS analysis was performed using a slight modification of the methods described elsewhere [24]. An odorant compound was identified in a urine sample using GC-MS (QP-2010A, Shimadzu, Japan) equipped with an automatic thermal desorption system (ATD 400, PerkinElmer, UK). Briefly, five drops of urine were dried at 60°C for 30 min, and these samples were absorbed into the tube at a speed of 100 mL per min for 5 min. The AT-1 capillary column applied was 60 m × 0.32 mm × 1.0 *μ*m, and the mass range was 20−350 m/z. The temperature ramp was in a programmed mode with an initial temperature of 35°C held for 10 min and then increased from 35°C to 120°C at a linear rate of 8°C/10 min, from 120°C to 180°C at a linear rate of 12°C/min, from 180°C to 230°C at a linear rate of 15°C/min, and held at 230°C for 10 min. The experimental mass spectra were compared with data stored in Wiley 229, NIST 21, and NIST 107 libraries to identify GC traces of the odorant compounds in urine metabolites.

### 2.6. Quantitative Real-Time (qRT)-PCR Analysis

The total RNA was purified from each foot tissue using an RNA Bee Solution (Tet-Test Inc., Friendswood, TX, USA) according to the manufacturer's instructions. After homogenization using a POLYTRON PT-MR 3100 D Homogenizer (Kinematica AG, Lucerne, Switzerland), the total RNAs were harvested from cell lysates by centrifugation at 10,000 × g for 15 min. The RNA concentration was determined using a Nano-300 Micro-Spectrophotometer (Allsheng Instruments Co. Ltd., Hangzhou, China). The total complementary DNA (cDNA) was synthesized against mRNA (4 *μ*g) using 200 units of Superscript II Reverse Transcriptase (Thermo Scientific, Wilmington, DE, USA). The specific DNA fragments for TMA dehydrogenase and *β*-actin were amplified from the mixture solution containing the cDNA template (1 *μ*L), along with 2× Power SYBR Green (6 *μ*L; Toyobo Life Science, Osaka, Japan) and specific primers (Table 1) using qRT-PCR. The cycle quantification value (*C*_*q*_) was calculated using Livak and Schmittgen's method described in a previous study [28].

### 2.7. Determination of Ammonia Concentration

The ammonia concentration in the urine sample was measured in the dirty bedding of the breeding cage using the GasAlertMicro 5 (BW Technologies by Honeywell, Calgary, Canada). While breeding the mice in the normal bedding cage for three, four, and five days, the ammonia concentration was measured daily in duplicate on the surface of the dirty bedding.

### 2.8. Histopathological Analysis

Foot samples were collected from the ICR mice of each subset group and then fixed in a 10% formalin solution for 48 h. After embedding the trimmed sole of the foot in paraffin blocks, tissue blocks were then sectioned into 4 *µ*m thick slices on slide glass. These sections were then stained with a hematoxylin and eosin (H&E) solution (Sigma-Aldrich; Merck KGaA, Darmstadt, Germany), followed by a microscopic examination of the histopathology features, at 400 × magnification.

### 2.9. Statistical Analysis

The statistical significance between the two- and 10-month-old ICR mice, as well as the vehicle-treated group and the EESJ-treated group, was analyzed using one-way analysis of variance (ANOVA, SPSS for Windows, Release 10.10, Standard Version; Chicago, IL, USA) followed by a Tukey post hoc *t*-test for multiple comparisons. All values are presented as the means ± SD, and a *p* value < 0.05 was considered significant.

## 3. Results

### 3.1. Composition and Deodorization Performance of EESJ

The phytochemical composition and deodorization performance of EESJ were first analyzed to confirm the potential based on our previous study. As shown in Figure 1(a), the high levels of TPC were detected (107.3 mg/g) compared with other bioactive components. The TTC and TFC concentrations were 65.7 mg/g and 28.6 mg/g, respectively. Moreover, EESJ produced a significantly high deodorizing performance against ammonia and acetic acid (91.2% and 54.8%, respectively). These results determine the high concentrations of phytochemical compounds present in EESJ and are based on their deodorization performance, indicating the potential of EESJ for application as a deodorant.

### 3.2. Identification of Novel Odor Marker Associated with Aging in the Urine of Old ICR Mice

**Figure 2:**
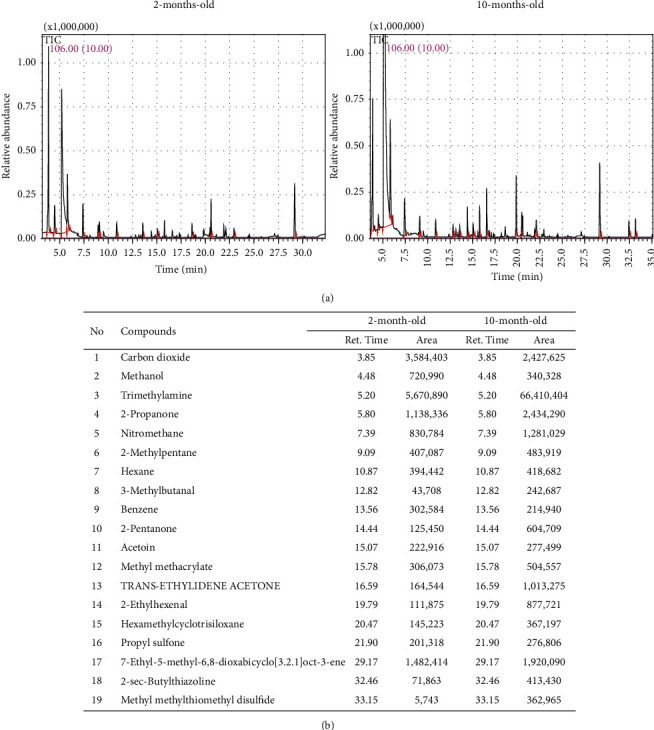
GC-MS total ion chromatograms and main volatile components in the urine of two- and 10-month-old ICR mice. (a) Chromatograms for urine samples. Each peak refers to the compounds mentioned in the bottom table. The volatile compounds were identified by comparing the MS spectrum and RIs of the components in EHF with the known authentic standards available in the NIST Library (2005). (b) Retention time and peak area of 19 compounds in the urine samples of two- and 10-month-old mice. GC-MS, gas chromatography-mass spectrometry; RIs, retention indices; EHF, extremely high frequency; NIST, National Institute of Standards and Technology.

**Figure 3:**
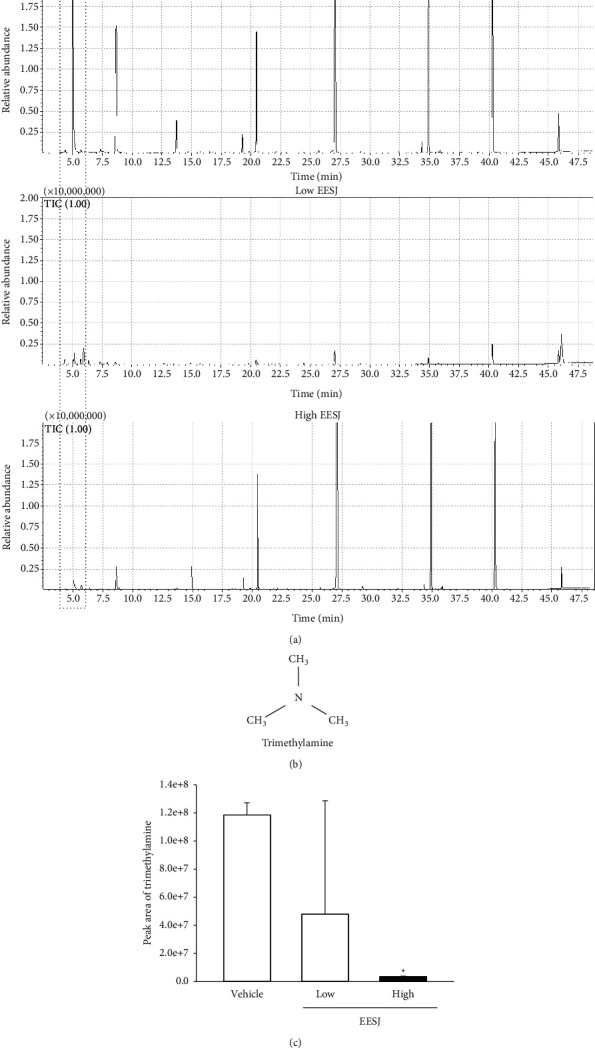
TMA concentration in the urine of EESJ-treated ICR mice. (a) The TMA concentration was measured in urine of subset groups using GC-MS analyses. (b) Chemical formula of TMA. (c) Relative concentration of peak area for TMA. Three to five mice per group were used to prepare the urine samples, and GC-MS was assayed in duplicate for each sample. Data are reported as the means ± SD. ^*∗*^*p* < 0.05 compared with the vehicle-treated group. TMA, trimethylamine; EESJ, 70% ethanol extract of *S. japonica*; GC-MS, gas chromatography-mass spectrometry.

The novel odor markers associated with aging were identified by comparative GC-MS analysis of the urine obtained from two- and 10-month-old ICR mice. Nineteen peaks were identified as candidate compounds, with a significant difference in the levels between the two groups. Of these, 16 compounds showed higher concentrations in the 10-month-old ICR mice compared with the two-month-old ICR mice, whereas three compounds showed lower levels in the same group. The TMA concentration was particularly high, with an 11.7-fold increase in the 10-month-old ICR mice compared with the two-month-old ICR mice (Figure 2). Therefore, TMA was selected as the novel odor marker associated with aging considering the above data and the ease of preparation that involves a reaction of ammonia and methanol employing a catalyst (Figure 3(b)) [29].

### 3.3. Alteration of the TMA Concentration in the Urine of 10-Month-Old ICR Mice after EESJ Administration

The changes in the TMA concentration were measured in the urine of 10-month-old ICR mice treated with LoEESJ and HiEESJ for four weeks to determine whether the oral administration of EESJ affects the TMA concentration. The TMA concentration decreased dose-dependently and significantly in the EESJ-treated groups compared with the vehicle-treated group (Figures 3(a) and 3(c)). These findings suggest that the oral administration of EESJ for four weeks decreases the TMA concentration in the urine of 10-month-old ICR mice.

### 3.4. Administration Effects of EESJ on the Ammonia Concentration in Dirty Bedding of 10-Month-Old ICR Mice

**Figure 4:**
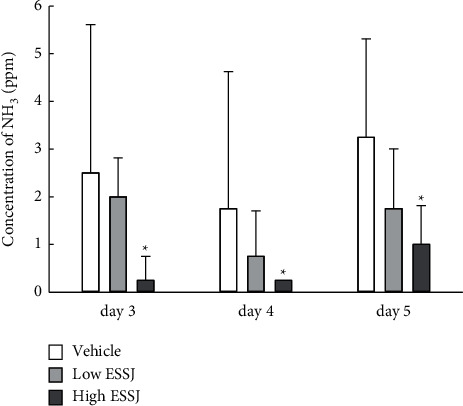
Ammonia concentration of dirty bedding. After treatment of EESJ for two weeks, the ammonia concentration was measured in dirty bedding obtained from a single ICR mouse, as described in Materials and Methods. Three to five mice per group were used to prepare the dirty bedding, and the ammonia concentrations were assayed in duplicate for each bedding. The data are reported as the means ± SD. ^*∗*^*p* < 0.05 compared with the vehicle-treated group. EESJ, 70% ethanol extract of *S. japonica*.

The changes on the concentration of ammonia was measured in dirty bedding obtained from the breeding cages of 10-month-old ICR mice administered with EESJ for four weeks to determine whether the decreased level of TMA is accompanied by an alteration of ammonia concentration in urine. The ammonia concentration was decreased dose-dependently and significantly in the EESJ-treated groups compared with the vehicle-treated group (Figure 4). These findings suggest that the suppression effects of EESJ on the TMA concentration in urine are associated with decreased ammonia concentrations in the dirty bedding of the breeding cages for 10-month-old ICR mice.

### 3.5. Effects of EESJ on the Histological Structure of Sweat Glands in Foot of 10-Month-Old ICR Mice

**Figure 5:**
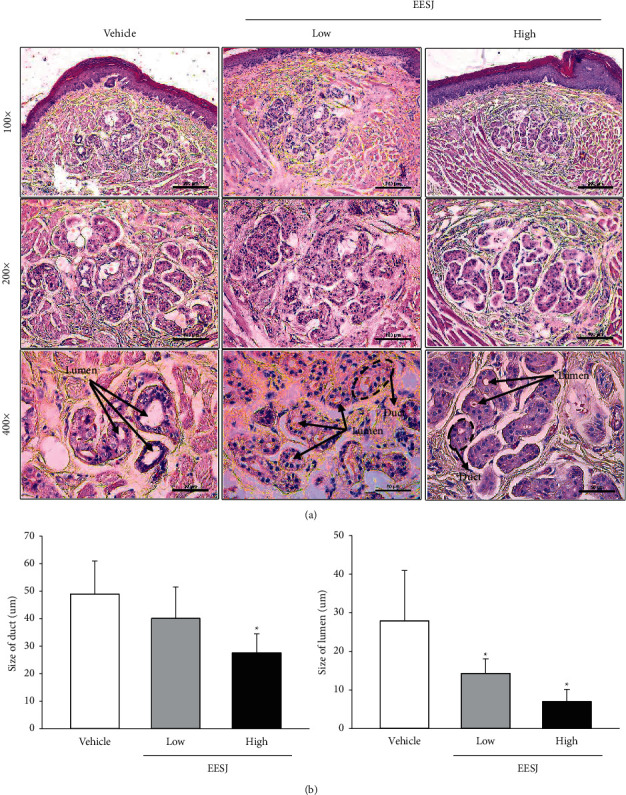
Histological structure of the sweat glands in mouse feet. (a) The histopathological structure of the sweat gland was observed in H&E-stained foot sections of EESJ-treated ICR mice at 100×, 200×, and 400× magnifications. (b) Size of the gland tubule and lumen in sweat glands was determined using the Leica Application Suite. Three to five mice per group were used to prepare the foot section, and the size of the gland tubule and lumen in sweat glands was counted in duplicate for each section. The data are reported as the means ± SD. ^*∗*^*p* < 0.05 compared with the vehicle-treated group. H & E, hematoxylin and eosin; EESJ, 70% ethanol extract of *S. japonica*.

**Figure 6:**
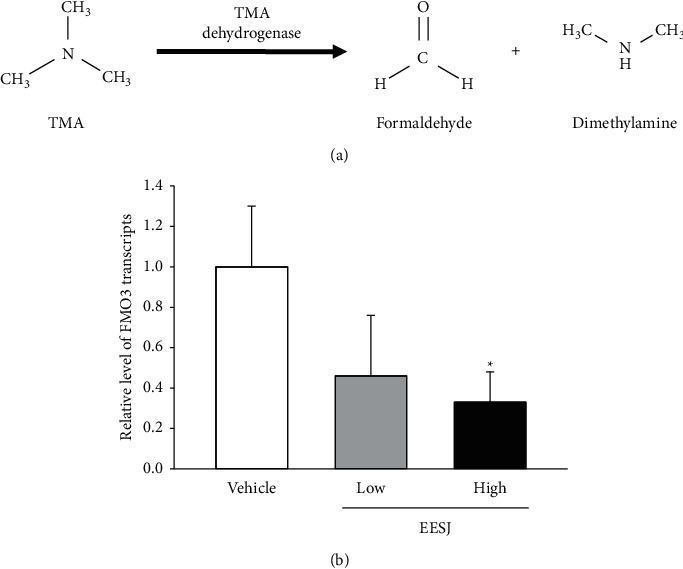
Transcription level of the genes for TMA metabolic enzymes. (a) Chemical reaction of TMA dehydrogenase. This enzyme catalyzes the oxidative demethylation of TMA to form formaldehyde and dimethylamine. (b) The transcript levels of TMA dehydrogenase were detected in the total mRNA of the foot tissue by qRT-PCR analysis using the specific primers. Three to five mice per group were used to prepare the total RNAs, and qRT-PCR was assayed in duplicate for each sample. The data are reported as the means ± SD. ^*∗*^*p* < 0.05 compared with the vehicle-treated group. FMO3, flavin-containing dimethylaniline monooxygenase 3; TMA, trimethylamine; EESJ, 70% ethanol extract of *S. japonica*; mRNA, messenger ribonucleic acid; qRT-PCR, quantitative real-time polymerase chain reaction.

Finally, this study investigated some associated changes in the histological structure of the sweat glands due to the deodorizing effects of EESJ in 10-month-old ICR mice. The changes in the histological structure were analyzed in the H&E-stained sections obtained from the feet of subset group mice. The number and size of the gland tubes in the sweat gland decreased significantly after the HiEESJ treatment compared with the vehicle-treated group. This alteration showed a dose-dependent decrease in the two EESJ-treated groups (Figure 5). Further investigation is needed to determine whether the above histological alterations in the mouse foot were accompanied by the expression regulation of the TMA metabolic enzyme. The expression level of the TMA dehydrogenase gene was significantly lower in the EESJ-treated group than in the vehicle-treated group (Figures 6(a) and 6(b)). These findings show that the deodorizing effects of EESJ contribute to the regulation of the sweat gland structure and TMA dehydrogenase gene expression in the feet of the 10-month-old ICR mice.

## 4. Discussion

Most conventional deodorants are linked to adverse health effects owing to their potentially toxic ingredients, including aluminum, octoxynol, and alkylphenol ethoxylates [30]. Numerous potentially deodorizing natural products have attracted considerable attention to prevent unnecessary exposure to harmful ingredients. Of these, a blend of essential oils and natural sweat-controlling ingredients might prevent odor-causing bacteria [30, 31]. This study evaluated the deodorizing effects of EESJ using a novel odor marker associated with aging in 10-month-old ICR mice to identify novel natural products with high deodorizing effects as ingredients for natural deodorants. The results from this study showed that TMA could be considered a novel odor marker linked to aging in old ICR mice. Furthermore, the data showed that the administration of EESJ in 10-month-old ICR mice had deodorizing effects, including decreased TMA concentration, transcriptional inhibition of TMA-related enzyme genes, suppression of ammonia, and changes in the number and structure of sweat glands. On the other hand, further studies will be needed to elucidate the molecular mechanism of EESJ and explain the deodorizing effects.

Thus far, few odor markers involved in age-related changes have been identified in the human body. The first marker is 2-nonenal, which is produced by the oxidative degradation of *ω*7 unsaturated fatty acid or lipid peroxides [13]. This compound was detected at high levels in older subjects during an analysis of the headspace by GC-MS. Simultaneously, the levels of *ω*7 unsaturated fatty acids and lipid peroxides increased with aging [13]. Other biomarkers identified as nonaxillary skin odorants associated with aging included dimethyl sulfone, benzothiazole, *α*-hexyl cinnamaldehyde, and nonanal. These volatile compounds were located abundantly in the backs and forearms of the elderly compared with younger donors [14]. In this study, TMA was identified as a novel odor marker associated with aging in the urine samples of 10-month-old ICR mice. This compound is produced by the catalytic reaction of ammonia and methanol [32]. At high concentrations, TMA has an ammonia-like odor, whereas it has a fishy odor at low concentrations [33]. Increased TMA concentrations were detected during the development of trimethylaminuria (fish odor syndrome) in the body, which is metabolized by the liver to trimethylamine N-oxide (TMAO), a possible proatherogenic substance [34, 35]. Moreover, TMA is possibly involved in developing the odor of several pathogenic infections, bad breath, and bacterial vaginosis in the human body [33].

Human body odors change continuously during life. In particular, elderly individuals have a specific body odor called kareishū, which is associated with “nursing home smell” and “old people smell” [15]. The chemical composition of body odor significantly changes in an age-dependent manner in humans and various animals [15, 36]. Some volatile compounds have been identified in urine samples by comparative analyses between young and old mice, even though the age of the animals differed in the experiments. Several age-dependent substances were first detected in the daytime urine of 12- and 23-month-old C57BL/6H mice [37]. Furthermore, the levels of 2-phenylacetamide and methylbutyric acids were observed to differ most prominently between adult (three- to 10-month-old) and aged (17- to 21-month-old) C57BL/6J-H-2^k^ mice [36]. Significant changes in the levels of 3, 4-dehydro-exo-brevicomin (DB), 2-sec-butyl-4, 5-dihydrothiazole (BT), 2-isopropyl-4, 5-dihydrothiazole (IT), and 6-hydroxy-6-methyl-3-heptanone were determined in the urine samples of aged (15- to 20-month-old) C57BL/6J mice [36]. Similar to previous studies, this study analyzed urine samples to identify an odor marker associated with aging. On the other hand, the animals used in these experiments were different from those used in previous studies. Unlike previous studies, 10-month-old ICR mice were used for the first time. These results suggested that TMA could be considered a potential odor marker obtained from the urine samples of aged mice.

The deodorization effects of natural products have been verified in both *in vitro* and *in vivo* models. The generation of ammoniacal nitrogen was suppressed by champignon extracts in chicken liver homogenate, while the levels of indoleacetic acid and tryptamine obtained in animal blood were reduced after the oral administration of these extracts [17]. Remarkable deodorization effects on malodor were measured in mushroom extracts and a supercritical hop extract of *H. lupulus* cones. The levels of two odor compounds (methylthiol and allyl thiol) were also decreased after treatment with mushroom extracts, but the axillary irritation scoring was applied in the deodorizing test of hop extract [18, 19]. A similar effect was observed with the methanolic extracts of camelina oil, sage extract, and immature pinecone extract [20–22]. This study examined the deodorizing effects of EESJ in aging mice. Significant changes in various parameters were detected, indicating the deodorizing effects in the EESJ-treated groups. These results were similar to previous studies, but the analysis factors were different. In particular, this study is the first to apply novel factors not employed in previous studies, such as the TMA concentration in urine, ammonia concentration in dirty bedding, and the structure of sweat glands. Therefore, these results provide the first evidence that the TMA level, ammonia concentration, and structure of the sweat glands are useful factors for evaluating the deodorizing effects.

## 5. Conclusion

The present *in vivo* studies investigated the deodorizing effects of EESJ using a novel odor marker associated with aging. Overall, these results provide the first scientific evidence that TMA can be considered an important marker for aging-associated odor. This study also provided evidence that EESJ can induce deodorization effects by suppressing the TMA concentration, ammonia level, and structure of sweat glands, as determined in 10-month-old ICR mice. Nevertheless, more studies will be needed to improve the understanding of the impending effects of a single compound, as well as the molecular mechanisms responsible for the deodorizing effects.

## Figures and Tables

**Table 1:** Primer sequences for qRT-PCR.

Primer name	Sequence (from 5′ to 3′)	Product size (bp)
TMA dehydrogenase		
Forward	AGA AAC CAA CCA TGG CAG TGA	77
Reverse	GCG TGC CTG CAG GTC AGT	

*β*-Actin		
Forward	TGG AAT CCT GTG GCA TCC ATG AAA C	349
Reverse	TAA AAC GCA GCT CAG TAA CAG TCC G	

## Data Availability

All data used to support the findings of this study are included in the article.
